# Hemoglobin glycation index predicts reduced mortality in critically ill patients with chronic kidney disease

**DOI:** 10.1016/j.clinsp.2025.100812

**Published:** 2025-10-29

**Authors:** Yangpei Peng, Wenwen Huang, Jie Wang

**Affiliations:** a Department of Nephrology, The Second Affiliated Hospital and Yuying Children’s Hospital of Wenzhou Medical University, Wenzhou, Zhejiang, China; b Department of Endocrinology, The Second Affiliated Hospital and Yuying Children’s Hospital of Wenzhou Medical University, Wenzhou, Zhejiang, China

**Keywords:** Hemoglobin Glycation index, Chronic kidney disease, Prognosis, Intensive care unit, Glycated hemoglobin A1c

## Abstract

**Background:**

Chronic Kidney Disease (CKD) is a worldwide health problem. Researchers have reported the close relation of the Hemoglobin Glycation Index (HGI) with metabolism, inflammation, and prognosis of disease. The prognostic value of HGI in CKD patients has not been assessed. This study aims to explore the association between HGI and mortality in critically ill patients with CKD.

**Methods:**

Data on critically ill patients diagnosed with CKD were extracted from the Medical Information Mart for Intensive Care-IV database. The HGI is the difference between an observed glycated hemoglobin A_1_c(HbA_1_c) and a predicted HbA1c obtained by inserting Fasting Plasma Glucose (FPG) into a regression equation describing the linear relationship between FPG and HbA1c in a reference population. The follow-up started on the patients’ first admission to the Intensive Care Unit (ICU). The primary outcome was 30-day mortality. 90-day and 365-day mortality were the secondary outcomes. Cox proportional hazards models were used to investigate the associations between HGI and mortality of CKD patients. Subgroup analyses were performed to assess the consistency of the association.

**Results:**

1,831 critically ill patients with CKD were included in the present study (64.1 % male, 60.2 % white, 71.93±12.72 years). For 30-day mortality, the Hazard Ratio (HR) value of the high-HGI group was 0.50 and 95 % Confidence Interval (95 % CI) was (0.39, 0.65) compared with the reference of the low-HGI group (p < 0.0001). When adjusted for age, gender and ethnicity, the adjusted HR (95 % CI) value of the high-HGI group was 0.53 (0.41, 0.68). When further adjusted for heart rate, diabetes mellitus, and SOFA score in Model II, the adjusted HR value of the high-HGI group was still statistically significant (HR = 0.57, 95 % CI: 0.44‒0.75, *p* < 0.0001). Similar results were also shown in the secondary outcomes of 90-day and 365-day mortality. Further subgroup analysis showed the above stable association between HGI and 30-day mortality of CKD patients.

**Conclusions:**

High level of HGI is associated with reduced short- and long-term all-cause mortality of critically ill patients with CKD. HGI can independently predict the prognosis of critically ill patients with CKD.

## Highlights

• First to evaluate the hemoglobin glycation index in critically ill CKD patients.
• A higher HGI is an independent predictor of reduced mortality in this population.
• The association remains significant after adjusting for relevant confounding factors.
• HGI is readily available and can help stratify risk in critically ill CKD patients.

## Introduction

Chronic Kidney Disease (CKD) refers to chronic structural and functional impairment of the kidney caused by various causes, with a history of kidney damage lasting more than 3-months. The increasing number of patients with CKD is a global concern.^1^^,^^2^ While the death rate from CKD is not as high as other serious diseases, such as cardiovascular disease, it has increased in recent decades.^3^ The treatment of established CKD is rather difficult, and the main aim is to delay the progression of kidney function. Many patients progress to end-stage renal disease and have to undergo dialysis or a kidney transplant. Despite aggressive management, the prognosis for CKD remains poor. In order to maximize the utilization of medical resources, it is necessary to search for prognostic markers of CKD. Previous research has shown a few promising biomarkers for CKD prognosis, including Red blood cell Distribution Width (RDW), Anion Gap (AG), Continuous Renal Replacement Therapy (CRRT),^4^ the Neutrophil-to-Lymphocyte Ratio (NLR),^5^ the Triglyceride Glucose index (TyG),^6^ etc.

The Hemoglobin Glycation Index (HGI), first proposed in 2002, is used to quantify how far an individual’s observed glycated Hemoglobin A_1_c(HbA_1_c) is above or below average compared to others with similar blood glucose concentrations.^7^ In recent years, more and more studies have reported the close relation between HGI and the prognosis of diseases, such as diabetes,^8^ cardiovascular diseases,^9^^,^^10^ liver disease,^11^^,^^12^ etc. To our knowledge, however, the prognostic value of HGI in CKD patients has not been evaluated. Therefore, we performed this study to explore the association between HGI and mortality of critically ill patients with CKD.

## Materials and methods

### Data resource

All data were extracted from a publicly available database, called the Medical Information Mart for Intensive Care-IV (MIMIC-IV).^13^ MIMIC-IV is developed by the computational physiology laboratory of the Massachusetts Institute of Technology (MIT). The database contains desensitization data on more than 50,000 critically ill patients admitted to Beth Israel Deaconess Medical Center (BIDMC) between 2008 and 2018. The data includes demographics, vital signs, laboratory indicators, medications, the scoring systems, etc. The establishment and use of this database were approved by the institutional review boards of MIT and BIDMC. The present study followed the STROBE Statement.

### Population selection criteria

Patients diagnosed with CKD were extracted. CKD was defined on the grounds of the Tenth Revision of the International Classification of Diseases (ICD-10) and was coded N18. CKD was defined as dipstick proteinuria or estimated Glomerular Rate (eGFR) < 60 mL/min/1.73 m^2^.

Patients with the following conditions were excluded: 1) Younger than 16-years of age at first admission; 2) The stay in ICU less than 48 hours; 3) Diagnosed with hematologic neoplasms, such as lymphoma, multiple myeloma, myelodysplastic syndrome and leukemia; 4) The loss of individual data more than 10 %; 5) Data value exceeded the mean ± 3-times the Standard Deviation (SD).

### Date collection

Baseline characteristics of included individuals, including demographics, vital signs, laboratory indicators, comorbidities, and the scoring system, were extracted within 24 hours on first admission to the ICU.

Demographics included age, gender and ethnicity. Vital signs included temperature, heart rate, respiratory rate, Diastolic Blood Pressure (DBP), Mean Blood Pressure (MBP) and Saturation of Percutaneous Oxygen (SPO_2_). Laboratory indicators included HbA_1_c, serum glucose, anion gap, serum potassium, hematocrit, hemoglobin, platelet counts and White Blood Cell (WBC) count. Comorbidities included Diabetes Mellitus (DM), Coronary Artery Disease (CAD), Congestive Heart Failure (CHF), Atrial Fibrillation (AF), stroke and chronic liver disease. The scoring system of Sequential Organ Failure Assessment (SOFA)^14^ was also recorded.

### Definition of exposure variables

**Fig. 1:**
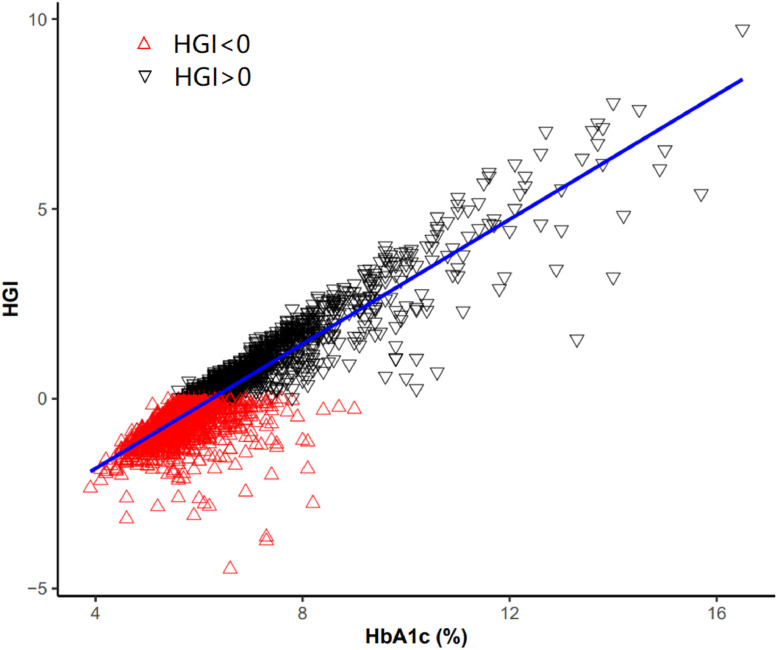
Correlation between HGI and HbA1c.

HGI is a linear regression residual. Firstly, a linear regression equation was established by incorporating glycated Hemoglobin (HbA1c) and Fasting Plasma Glucose (FPG) levels (The predicted HbA1c = -0.0075FPG+5.5452). The HGI was subsequently calculated based on the difference between the predicted and observed HbA1c levels (HGI = observed HbA1c-predicted HbA1c).^15^ The correlation between HGI and HbA1c was shown in Fig. 1.

### Follow-up and outcomes

Follow-up began when patients were first admitted to the ICU. The primary outcome was all-cause mortality within 30 days after admission. The secondary outcomes were 90-day and 365-day mortality.

### Statistical analysis

A linear relationship between HGI and 30-day mortality was identified using multivariate Restricted Cubic Spline (RCS) analysis. Enrolled patients were divided into two groups: the low-HGI group (HGI < -0.44) and the high-HGI group (HGI ≥ -0.44).

Continuous data were expressed as mean ± Standard Deviation (SD), compared by the variance analysis or the Kruskal-Wallis test^16^ between groups. Categorical data were expressed as frequency (percentage), compared by chi-square test^17^ or Fisher’s exact test.^18^ Cox proportional hazards models^19^ were applied to investigate the associations between HGI and outcomes of CKD patients. Each outcome was respectively analyzed by three models: Crude model didn’t adjust for confounders; Model I adjusted for age, gender and ethnicity; Model II adjusted for age, gender, ethnicity, heart rate, DM, and SOFA. These confounders were selected based on their relevance to the outcome or the presence of mutations greater than 10 %.^20^. The low-HGI group was defined as the reference. The results were expressed as Hazard Ratio (HR) with 95 % Confidence Interval (95 % CI). In addition, subgroup analyses were performed to assess the consistency of the association between HGI and 30-day mortality of CKD patients.

A double-tailed p-value < 0.05 was deemed statistically significant. The data processing in this study was achieved by *R* software version 4.2.

## Results

### Baseline characteristics

**Fig. 2:**
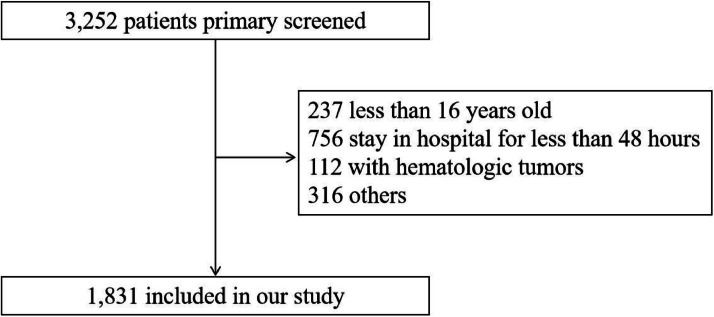
Flow chart of the included population.

A total of 1,831 critically ill patients with CKD were included in this study. The flow chart of the included population was shown in Fig. 2. The included patients had a mean (±SD) age of 71.93 (±12.72) years. Males accounted for 64.1 % and the white population accounted for 60.2 % of the population. The included patients were divided into two groups: 914 in the low-HGI group and 917 in the high-HGI group. The results of baseline characteristics are shown in Table 1. Patients in the high-HGI group were more likely to be younger and have a lower anion gap than the low-HGI group. Patients with a higher level of HGI were more likely to have DM, CAD, but less likely to have AF and chronic liver disease. Also, these patients showed a significantly lower SOFA score.

**Table 1:** Baseline characteristics of the study population.

Variables	HGI		p-value
	Low	High	
N	914	917	
HGI	-1.11 ± 0.68	1.10 ± 1.54	<0.001
Age	72.97 ± 13.13	70.90 ± 12.32	<0.001
Gender, n ( %)			0.848
Female	326 (35.67 %)	331 (36.10 %)	
Male	588 (64.33 %)	586 (63.90 %)	
Ethnicity, n ( %)			0.033
White	575 (62.91 %)	527 (57.47 %)	
Black	108 (11.82 %)	139 (15.16 %)	
Other	231 (25.27 %)	251 (27.37 %)	
LOS_ICU	4.48 ± 5.58	3.97 ± 5.81	<0.001
Vital signs			
Temperature,°C	36.74 ± 0.47	36.76 ± 0.45	0.940
Heart rate, beats/min	80.97 ± 14.15	80.67 ± 12.59	0.737
Respiratory rate, beats/minute	18.84 ± 3.24	18.55 ± 3.16	0.104
DBP, mmHg	62.11 ± 13.40	60.93 ± 11.64	0.288
MBP, mmHg	79.35 ± 13.33	78.07 ± 11.23	0.148
SPO_2_, %	97.16 ± 1.88	97.25 ± 1.69	0.614
Laboratory parameters			
HbA_1_c, %	5.62 ± 0.62	7.89 ± 1.88	<0.001
Glucose, mg/dL	157.49 ± 105.75	166.39 ± 102.88	0.017
Anion gap, mmoL/L	14.11 ± 3.92	13.33 ± 3.54	<0.001
Serum potassium, mmoL/L	4.13 ± 0.61	4.14 ± 0.57	0.485
Hematocrit	28.79 ± 6.58	29.22 ± 6.31	0.140
Hemoglobin, g/dL	9.43 ± 2.22	9.59 ± 2.11	0.091
Platelets, 10^9^/L	173.59 ± 92.96	178.44 ± 84.30	0.130
WBC count, 10^9^/L	10.26 ± 5.20	9.77 ± 4.26	0.360
Comorbidities, n ( %)			
DM	155 (16.96 %)	440 (47.98 %)	<0.001
CAD	454 (49.67 %)	545 (59.43 %)	<0.001
CHF	472 (51.64 %)	492 (53.65 %)	0.389
AF	426 (46.61 %)	353 (38.50 %)	<0.001
Stroke	125 (13.68 %)	125 (13.63 %)	0.978
Chronic liver disease	53 (5.80 %)	33 (3.60 %)	0.026
Scoring system			
SOFA	5.92 ± 3.31	5.45 ± 2.95	0.013
Death, n ( %)			
30-Day	174 (19.04 %)	90 (9.81 %)	<0.001
90-Day	231 (25.27 %)	131 (14.29 %)	<0.001
365-Day	306 (33.48 %)	214 (23.34 %)	<0.001

Continuous data were presented as $\bar{x}\pm \text{SD}$ and categorical data are presented as n ( %).

HGI, The Hemoglobin Glycation Index; N, Number; LOS_ICU, Length of Stay in Intensive Care Unit; DBP, Diastolic Blood Pressure; MBP, Mean Blood Pressure; SPO_2_, Saturation of Percutaneous Oxygen; HbA_1_c, Hemoglobin A_1_c; WBC, White Blood Cell; DM, Diabetes Mellitus; CAD, Coronary Artery Disease; CHF, Congestive Heart Failure; AF, Atrial Fibrillation; SOFA, Sequential Organ Failure Assessment.

### HGI and mortality of CKD patients

The results of Cox proportional hazards regression were presented in Table 2. For 30-day mortality, the HR (95 % CI) value of the high-HGI group was 0.50 (0.39, 0.65) compared with the reference of low-HGI group (p < 0.0001). When adjusted for age, gender and ethnicity in Model I, the adjusted HR (95 % CI) value of the high-HGI group was 0.53 (0.41, 0.68). When further adjusted for HR, DM and SOFA in Model II, the adjusted HR value of the high-HGI group was still statistically significant (HR = 0.57, 95 % CI: 0.44‒0.75, p < 0.0001). Similar results were also shown in the secondary outcomes of 90-day and 365-day mortality. The adjusted HR (95 % CI) values of the high-HGI group were 0.58 (0.46, 0.73) for 90-day mortality and 0.69 (0.58, 0.84) for 365-day mortality.

**Table 2:** The association between HGI and mortality of CKD patients.

	Non-adjusted		Model I		Model II	
	HR (95 % CI)	p-value	HR (95 % CI)	p-value	HR (95 % CI)	p-value
**30-day mortality**						
Low	1.0		1.0		1.0	
High	0.50 (0.39, 0.65)	<0.0001	0.53 (0.41, 0.68)	<0.0001	0.57 (0.44, 0.75)	<0.0001
**90-day mortality**						
Low	1.0		1.0		1.0	
High	0.54 (0.43, 0.67)	<0.0001	0.57 (0.46, 0.71)	<0.0001	0.58 (0.46, 0.73)	<0.0001
**365-day mortality**						
Low	1.0		1.0		1.0	
High	0.65 (0.54, 0.77)	<0.0001	0.68 (0.57, 0.81)	<0.0001	0.69 (0.58, 0.84)	0.0001

HR, Hazard Ratio; CI, Confidence Interval.

Models I and II were derived from Cox proportional hazards regression models: Model I covariates were adjusted for age; gender; ethnicity; Model II covariates were adjusted for age; gender; ethnicity; HR; DM; SOFA.

### Subgroup analyses

Subgroup analyses of the association between HGI and 30-day mortality are shown in Table 3. There were no differences between groups in age, gender, ethnicity, CAD, CHF, AF, and stroke. The differences were shown in the subgroups of DM and SOFA score. Patients without a history of DM showed a significantly low risk of 30-day mortality for the high-HGI group (HR = 0.48; 95 % CI [0.35, 0.67]). For patients with a history of DM, however, HR (95 % CI) for the high-HGI group was 0.65 (0.39, 1.07), and the difference was not statistically significant. For the SOFA score, patients with a SOFA score of 5‒18 showed a significantly lower risk of 30-day mortality for the high-HGI group (HR = 0.48; 95 % CI [0.35, 0.64]). For patients with a SOFA score of 0‒4, the difference was not statistically significant (HR = 0.64; 95 % CI [0.39, 1.03]).

**Table 3:** Subgroup analyses of the association between the HGI and 30-day mortality.

Subgroups	n	HGI		p-value
		Low	High	
Vital signs				
Age, year				
24‒74	915	1.0	0.39 (0.25, 0.61)	<0.0001
74‒98	916	1.0	0.61 (0.45, 0.84)	0.0021
Gender				
Female	657	1.0	0.45 (0.30, 0.69)	0.0002
Male	1174	1.0	0.53 (0.39, 0.74)	0.0001
Ethnicity				
White	1102	1.0	0.50 (0.35, 0.70)	<0.0001
Black	247	1.0	0.41 (0.20, 0.86)	0.0185
Other	482	1.0	0.55 (0.35, 0.85)	0.0078
Comorbidities				
DM				
No	1236	1.0	0.48 (0.35, 0.67)	<0.0001
Yes	595	1.0	0.65 (0.39, 1.07)	0.0922
CAD				
No	832	1.0	0.63 (0.45, 0.89)	0.0091
Yes	999	1.0	0.41 (0.28, 0.60)	<0.0001
CHF				
No	867	1.0	0.39 (0.26, 0.60)	<0.0001
Yes	964	1.0	0.58 (0.42, 0.81)	0.0012
AF				
No	1052	1.0	0.52 (0.36, 0.76)	0.0006
Yes	779	1.0	0.52 (0.37, 0.74)	0.0003
Stroke				
No	1581	1.0	0.52 (0.39, 0.70)	<0.0001
Yes	250	1.0	0.44 (0.26, 0.74)	0.0018
Score systems				
SOFA				
0‒4	758	1.0	0.64 (0.39, 1.03)	0.0680
5‒18	1073	1.0	0.48 (0.35, 0.64)	<0.0001

HGI, The Hemoglobin Glycation Index; DM, Diabetes Mellitus; CAD, Coronary Artery Disease; CHF, Congestive Heart Failure; AF, Atrial Fibrillation; SOFA, Sequential Organ Failure Assessment.

## Discussion

HbA1c, a traditional glycemic monitoring metric, is widely used in clinical practice. However, only 60 %‒80 % of HbA1c reflects average blood glucose levels, with the remaining 20 %‒40 % variation attributed to factors such as age, genetic variation, red blood cell longevity, and race.^21^ Additionally, in CKD patients, the reliability of HbA1c is further reduced due to CKD-related abnormalities affecting red blood cell turnover, such as suppressed erythropoiesis or a shortened red blood cell lifespan. Changes in hemoglobin conversion and carbamylation associated with uremia also interfere with the measurement of HbA1c. HGI is a biomarker of population variation in HbA1c due to factors other than blood glucose concentration.^22^ HGI quantifies the magnitude and direction of inter-individual variation in HbA1c based on the difference between an observed HbA1c and a predicted HbA1c. Derived from FPG and HbA_1_c, HGI appears to be more reliable.

Previous research has reported that HGI is closely related to metabolism,^23^ inflammation^24^^,^^25^ and incidence of disease.^26^, ^27^, ^28^ A growing number of studies have recently explored the prognostic role of HGI in diseases, primarily in cardiovascular disease, but also in sepsis, liver disease, etc. Zhao, L. et al.^29^ performed a cohort study of an American metabolic syndrome population of more than 8,000 people. They highlighted a U-shaped association of HGI with all-cause and cardio-cerebrovascular mortality in the above population. He, A. et al.^30^ have found that there is a significant association between HGI and all-cause mortality in patients with sepsis, and patients with higher HGI values had a higher risk of death.

The present study focused specifically on CKD patients, confirming the predictive role of HGI in the prognosis of CKD patients. The results indicate that high level of HGI is associated with decreased short-term and long-term mortality of patients with CKD. Further subgroup analysis showed good stability in the relationship between HGI and mortality in patients with CKD. Low HGI has previously been reported to be relevant to adverse clinical outcomes. Shangguan, Q. et al.^10^ reported in their study, using NHANES data, that low HGI was significantly associated with increased all-cause mortality in people with high blood pressure. In a study^31^ that also used the MIMIC-IV database, a low HGI was also found to increase the 365-day mortality in patients with critical coronary artery disease. Recently, Zhao, M. et al.^11^ also proposed an increased risk of all-cause mortality with a low level of HGI in patients with metabolic dysfunction-associated steatotic liver disease.

The possible mechanisms by which HGI affects all-cause mortality are as follows. First of all, HGI is the difference between an observed HbA1c and a predicted HbA1c. The actual HbA1c levels of individuals with HGI significantly below zero were significantly lower than the average HbA1c levels observed in populations with similar FPG levels. Due to the inaccurate estimate of HbA1c levels, patients with low HGI may be considered to have good glycemic control. The glucose of these patients may not be properly managed, leading to further deterioration of the condition. Secondly, low HGI may serve as an indicator of frequent hypoglycemia, which has already been confirmed to be associated with mortality of patients with vascular events,^32^^,^^33^ sepsis,^34^ hemodialysis.^35^ In CKD patients, hypoglycemia occurs more easily even in the absence of diabetes, for impaired renal gluconeogenesis, reduced renal degradation of insulin, co-existing comorbidities (such as protein-energy wasting and diabetic gastroparesis), as well as inhibition of hepatic glucose output and stimulation of insulin secretion by uremic metabolites.^36^ Moreover, HGI has also been reported to be associated with inflammation. Shuqian Liu et al. have proposed in their study that HGI reflects the effects of inflammation on HbA1c in a nondiabetic population of American adults.^24^ Inflammation may play a role in the relationship between HGI and the mortality of CKD patients. However, the above is only the authors’ speculation, and the exact mechanism by which HGI is associated with all-cause mortality still needs further investigation.

As a stable and cost-effective prognostic biomarker, HGI can help clinicians quickly identify high-risk patients and make better medical decisions in clinical practice. The present study is the first to evaluate the prognostic value of HGI in critically ill patients with CKD. However, it does have some limitations. Firstly, this is a retrospective research of a single center’s public database, which inevitably has a selection bias. Further prospective researches are needed. Secondly, a relatively small sample size of this study suggests research with a larger capacity is needed in the future. Thirdly, the specific etiology of CKD is unknown, making it uncertain whether HGI is meaningfully associated with different etiologies.

## Conclusions

High level of HGI is associated with reduced short- and long-term mortality of critically ill patients with CKD. HGI, a readily available biomarker, can independently predict the prognosis of critically ill patients with CKD. These conclusions need to be further confirmed by prospective studies with larger sample sizes.

## Authors’ contributions

Yangpei Peng: Conceptualization; methodology; resources; data curation; writing-original draft preparation.

Wenwen Huang: Methodology; software; formal analysis; data curation.

Jie Wang: Methodology; software; conceptualization; visualization; validation; writing-reviewing and editing.

## Funding

None.

## Human ethics

All de-identified data were extracted from the publicly available MIMIC-IV database. The establishment and use of this database were approved by the institutional review boards of MIT and BIDMC. The research was conducted in accordance with the Declaration of Helsinki.

## Consent to participate

Not applicable.

## Consent for publication

Not applicable.

## Data availability statements

The datasets generated and/or analyzed during the current study are available in the open MIMIC-IV database[https://mimic.mit.edu].

## Declaration of competing interest

The authors declare no conflicts of interest.
